# *In vitro* and *in vivo* effects of standardized extract and fractions of *Phaleria macrocarpa* fruits pericarp on lead carbohydrate digesting enzymes

**DOI:** 10.1186/1472-6882-13-39

**Published:** 2013-02-20

**Authors:** Rabyah B Ali, Item J Atangwho, Navneet Kuar, Mariam Ahmad, Roziahanim Mahmud, Mohd Z Asmawi

**Affiliations:** 1 School of Pharmaceutical Sciences, Universiti Sains Malaysia, Minden 11800, Penang, Malaysia; 2 Department of Biochemistry, College of Medical Sciences, University of Calabar, P. M. B. 1115, Calabar, Nigeria

**Keywords:** α-Glucosidase, α-Amylase, Peak blood glucose, Postprandial hyperglycaemia, Type 2 diabetes mellitus, *Phaleria macrocarpa*

## Abstract

**Background:**

One vital therapeutic approach for the treatment of type 2 diabetes mellitus is the use of agents that can decrease postprandial hyperglycaemia by inhibiting carbohydrate digesting enzymes. The present study investigated the effects of bioassay-guided extract and fractions of the dried fruit pericarp of *Phaleria macrocarpa*, a traditional anti-diabetic plant, on α-glucosidase and α-amylase, in a bid to understand their anti-diabetic mechanism, as well as their possible attenuation action on postprandial glucose increase.

**Methods:**

Methanol extract (ME), obtained by successive solvent extraction, its most effective liquid-liquid n-butanol fraction (NBF) and the flash column chromatographic sub-fraction (SFI), were evaluated for *in vitro* α-glucosidase (yeast) and α-amylase (porcine) activity inhibition. Furthermore, confirmatory *in vivo* tests were carried out in streptozotocin-induced diabetic rats (SDRs) using oral glucose, sucrose and starch tolerance tests.

**Results:**

At the highest concentration employed (100 μg/ml), NBF showed highest inhibition against α-glucosidase (75%) and α-amylase (87%) *in vitro* (IC_50_ = 2.40 ± 0.23 μg/ml and 58.50 ± 0.13 μg/ml, respectively) in a dose-dependent fashion; an effect found to be about 20% higher than acarbose (55%), a standard α-glucosidase inhibitor (IC_50_ = 3.45 ± 0.19 μg/ml). The ME and SFI also inhibited α-glucosidase (IC_50_ = 7.50 ± 0.15 μg/ml and 11.45 ± 0.28 μg/ml) and α-amylase (IC_50_ = 43.90 ± 0.19 μg/ml and 69.80 ± 0.25 μg/ml), but to a lesser extent. In *in vivo* studies with diabetic rats, NBF and SFI effectively reduced peak blood glucose (PBG) by 15.08% and 6.46%, and the area under the tolerance curve (AUC) by 14.23% and 12.46%, respectively, after an oral sucrose challenge (*P* < 0.05); thereby validating the observed *in vitro* action. These reduction effects on PBG and AUC were also demonstrated in glucose and starch tolerance tests, but to a lesser degree.

**Conclusions:**

These findings reveal that *P*. *macrocarpa* can attenuate hyperglycaemia in both *in vitro* and *in vivo* conditions by potently inhibiting carbohydrate hydrolysing enzymes, making it a viable plant for sourcing natural compounds for the management of type 2 diabetes mellitus.

## Background

Plants have been exemplary sources of medicine since ancient times. They have played key roles in traditional health care systems and on the basis of this, have become a significant percentage of allopathic and modern drugs in many nations of the world [1,2]. Medicinal plants are therefore used as modern alternatives to orthodox medicines or as complementary products to maintain health or treat aspects of diseases, particularly where conventional medication has had only limited success [3].

Diabetes is one such disease that has been managed with only limited success by “Western” medicine. Conventional effort at better management of diabetes has been disappointing and the control of blood glucose level remains unsatisfactory, as reflected in daily increases in morbidity and mortality rates [4]. Consequently, the current focus for appropriate anti-diabetic agents is herbal medicine. There is, however, a need for more in-depth investigation to confirm and advocate the benefits of these plants over existing therapies, including elucidation of their mechanism(s) of action and therapeutic effects, as the anti-diabetic evidence is often anecdotal [5].

*Phaleria macrocarpa* (Scheff) Boerl (Thymelaceae), a shrub known locally as Mahkota Dewa, literally translated as “God’s Crown”, has for centuries been used by the native Indonesians and the lower course of Malaysia to combat diabetes, liver diseases, vascular problems, cancer, and high blood pressure [6]. The parts of *P*. *macrocarpa* that are used for medicinal treatments are the stems, leaves and fruits. Although the works of Triastu and Choi [7] and Triastu *et al*. [8] on oxidative stress protection in alloxan diabetes suggest scientific validation of its anti-diabetic activity, there are no reports, to our knowledge, on the detailed anti-diabetic mechanism of *P*. *macrocarpa*. Earlier exploratory studies with young and old fruit extracts of *P*. *macrocarpa* on non-diabetic rats [9], and later with young and old leaf extracts [10] suggested that *P*. *macrocarpa* possessed possible α-glucosidase inhibitory activity.

It was therefore necessary to conduct a detailed study with the aim to understand at least in part, the anti-diabetic mechanism of *P*. *macrocarpa* as it relates to inhibition of carbohydrate hydrolysis since drugs with this property can circumvent postprandial hyperglycaemic risk in diabetes. *In vitro* studies are relatively simple, precise and most suitable when a large number of compounds or fractions are to be tested, and are also used in mechanistic elucidation [11]. However, a confirmatory *in vivo* test is also necessary to substantiate positive *in vitro* results, since positive α-glucosidase inhibitory action may not always correlate with *in vivo* actions [12]. Accordingly, the present study employed both *in vitro* and *in vivo* test procedures to evaluate the effect of *P*. *macrocarpa* on carbohydrate digesting enzymes. Moreover, the studies also followed a systematized drug discovery program of bioassay-guided extraction and test procedures. An activity-guided approach is indispensable in natural product discovery or standardization of herbal products for use as alternatives and/or complements to conventional medicines.

The present study evaluated the *in vitro* inhibition of yeast α-glucosidase (EC 3.2.1.20) and porcine pancreatic α-amylase (EC 3.2.1.1) activities of the active crude extract, fraction and sub-fraction of *P*. *macrocarpa* identified earlier from activity-guided hypoglycaemic and anti-hyperglycaemic tests carried out in our laboratory [13,14]. A confirmatory *in vivo* study was also conducted in non-diabetic and streptozotocin-induced diabetic rats.

## Methods

### Preparation of plant extracts and fractions

Fruits of *Phaleria macrocarpa* were collected from Kepala Batas, Seberang Perai, Pulau Pinang, Malaysia in August, 2010. They were identified by Mr. V. Shunmugam a/l Vellosamy and a voucher specimen of the plant (voucher number 11259) was deposited in the herbarium unit, School of Biological Sciences, Universiti Sains Malaysia. The pericarps of the fruits were sliced, dried, and powdered using a milling machine. About 2,400 g were successively extracted with petroleum ether and methanol using Soxhlet apparatus (40°C) for 48 h each. Thereafter, the residue from the methanol extraction after complete drying was extracted with water by maceration at 60°C for 24 hours. Extraction with each solvent was done in triplicate and the extracts obtained were filtered with Whatman No. 1 filter paper and concentrated *in vacuo* by rotary evaporation (Buchi Labortechnik, Flawil, Switzerland). The concentrated extracts were finally lyophilized to obtain 73.6 g (3.06%), 445.36 g (18.55%) and 146 g (6.08%) each of dried petroleum ether extract (PEE), methanol extract (ME) and water extract (WE), respectively. Earlier results from hypoglycaemic and anti-hyperglycaemic tests with these extracts showed that the methanol extract was the most effective in lowering blood glucose [13], and thus this alone was used in the present study.

### Successive liquid-liquid fractionation of the methanol extract

The methanol extract of *Phaleria macrocarpa* was fractionated with polarity graded solvents in separating funnels. In brief, 110 g of the methanol extract was first extracted with 3 × 360 ml of chloroform-water (6:5). The combined chloroform fractions were dried with anhydrous sodium sulphate and further concentrated in a rotary evaporator. The aqueous layer was extracted with 3 × 250 ml ethyl acetate and the combined ethyl acetate fractions were concentrated as above. Finally, the aqueous layer was extracted with n-butanol 5 × 250 ml and the combined n-butanol fraction was concentrated as well as the remaining aqueous fraction. The concentrated fractions were thereafter freeze-dried to obtain 18 g (5.45%), 24.3 g (7.36%), 110.1 g (33.3%) and 89.1 g (27%) of chloroform (CF), ethyl acetate (EAF), n-butanol (NBF) and aqueous (AF) fractions, respectively. Previous results from the hypoglycaemic and anti-hyperglycaemic tests with these fractions revealed that the n-butanol fraction was the most effective [14], and hence this alone was selected for the present investigation.

### Fractionation of the active n-butanol fraction by dry-column flash chromatography

A chromatographic glass column (27 × 5 cm) used in the separation was gently loaded with 100 g of silica gel (Merck, 7730) in 300 ml petroleum ether. The silica was carefully packed by applying vacuum suction, and a levelled and well-compacted bed yield was ensured. The n-butanol fraction (12 g) was pre-adsorbed onto the adsorbent (silica gel, 200-400 mesh) by first dissolving in methanol (100 ml), followed by addition of the silica gel (24 g). The mixture was evaporated to dryness using a rotary evaporator, and the resultant dried extract-adsorbent mixture was then loaded onto the top of the already packed column evenly by applying suction. The column was first eluted with 2 × 300 ml 100% chloroform, followed serially by 2 × 300 ml chloroform-methanol in graded ratios: (9:1), (8:2), (7:3), (6:4), (5:5), (4:6), (3:7), (2:8), (1:9), (0:10) and finally with chloroform-methanol-water (7:13:2). Fractions were collected in a fixed volume and examined with thin layer chromatography using n-butanol-acetic acid-water (4:1:5) as the mobile phase. Fractions with similar profiles were pooled together offering two sub-fractions namely SFI and SFII which were freeze-dried to obtain 20 g (40%) and 8 g (16%) respectively. When these sub-fractions were subjected to hypoglycaemic and anti-hyperglycaemic screening, sub-fraction I was found to be the most active [14], therefore it was used for the current α-glucosidase and α-amylase inhibition tests.

### Animals

Healthy male Sprague Dawley (SD) rats weighing 200-250 g obtained from the Animal Research and Service Centre, Universiti Sains Malaysia (USM) were used for this study. These were housed in the Animal Transit Room, School of Pharmaceutical Sciences, USM. They were allowed free access to food (standard laboratory chow, Gold Coin Sdn. Bhd., Malaysia) and tap water. The animals were maintained according to accepted international and national guidelines and the procedure for this experiment approved by the Animal Ethics Committee of Universiti Sains Malaysia, Penang, Malaysia (AECUSM). Diabetes was induced in the rats by intra-peritoneal injection of 65 mg/kg b.w. of streptozotocin (Sigma, St Louis, MO, USA), after an overnight fast [15]. Seventy-two (72) hours after, their blood glucose levels were measured using the Accu-check Advantage II Glucose meter (Roche Diagnostics Co., USA) and rats with fasting blood glucose ≥ 15 mmol/L were considered diabetic and included in the study. The effects of *P*. *macrocarpa* extract, fraction and sub-fraction on oral carbohydrate tolerance (starch, sucrose and glucose), an indirect measure of α-glucosidase and α-amylase activities, were evaluated in non-diabetic rats (NDRs) and streptozotocin diabetic rats (SDRs) categorized into groups as shown below. Acarbose, a conventional α-glucosidase inhibitor, was used as a positive control in the two sets of experiments.

### *In vitro* α-glucosidase (EC 3.2.1.20) inhibition study

The assay was performed using our earlier procedure [11]. In brief, 50 μl of 4 graded concentrations (100 μg/ml, 50 μg/ml, 25 μg/ml, 12.5 μg/ml) each of sample (extract/fraction/sub-fraction) and acarbose, the positive control, were suspended in 100 μl of 0.1 M phosphate buffer (pH 6.9) containing yeast α-glucosidase (Sigma Aldrich Chemical Co, USA) solution (1.0 U/L) and pre-incubated in a 96-well microplate at 25°C for 10 min. After pre-incubation, 50 μl of 5 mM p-nitrophenyl-α-D-glucopyranoside solution (the enzyme substrate), in 0.1 M phosphate buffer (pH 6.9) was added to each well. An equivalent volume (50 μl) of buffer solution was added to the blank or control in place of the extract. The reaction mixtures were incubated at 25°C for 5 min. The absorbance of the reaction mixtures before and after substrate incubation was measured at 405 nm on a micro-plate reader (Power Wave Biotek Instrument Inc, USA). The α-glucosidase inhibitory activity was calculated from the difference in the two absorbances and expressed as % inhibition as follows:

$$\%\;\text{Inhibition}=\frac{A\;540\;\left(\text{control}\right)-A540\;\left(\text{extract}\right)}{A540\;\left(\text{control}\right)}\times 100$$

The experiment was performed in triplicate. The IC_50_, i.e. the concentration of the extract/fraction/acarbose resulting in 50% inhibition of the enzyme was calculated by regression analysis.

### *In vitro* α-amylase (EC 3.2.1.1) inhibition study

A total of 500 μl of each sample and 500 μl of 0.02 M sodium phosphate buffer (pH 6.9) containing porcine α-amylase solution (0.5 mg/ml) were incubated at 25°C for 10 min. After pre-incubation, 500 μl of 1% starch solution in 20 mM sodium phosphate buffer (pH 6.9) was added to each test tube. The reaction mixtures were then incubated at 25°C for 10 min and thereafter stopped by addition of 1 mL of 3,5-dinitrosalicylic acid (DNS) colour reagent. The test tubes were then incubated in boiling water for 5 min and then cooled to room temperature. After dilution of the reaction mixtures with 10 ml of distilled water, the absorbance was measured at 540 nm. Acarbose was used as the positive control. The inhibition activity was calculated as follows:

$$\%\;\text{Inhibition}=\frac{A\;540\;\left(\text{control}\right)-A540\;\left(\text{extract}\right)}{A540\;\left(\text{control}\right)}\times 100$$

Control incubations representing 100% enzyme activity were carried out in a similar fashion by replacing the plant extract/fraction with vehicle (500 μl DMSO and distilled water). For the blank incubation, the enzyme solution was replaced with distilled water and the same procedure was followed as above. Separate incubations conducted for the reaction of t = 0 min was performed by adding samples to the DNS solution immediately after addition of the enzyme. The experiment was also performed in triplicate and the IC_50_, i.e. the concentration of the extract/fraction/acarbose resulting in 50% inhibition of the enzyme was calculated by regression analysis.

### Confirmatory *in vivo* studies in non-diabetic rats (NDRs)

#### Starch tolerance test

In this test, 30 overnight-fasted non-diabetic rats divided into five groups of six each were respectively treated (p.o.) with ME (1 g/kg), NBF (1 g/kg), SFI (1 g/kg), acarbose (positive control, 10 mg/kg), and distilled water (negative control). Ten minutes after, the rats were administered starch (3 g/kg body weight) orally and blood was collected via tail puncture for blood glucose estimation before (0 min) and at 30, 60 and 120 minutes after starch treatment [16]. The recorded blood glucose concentrations peak blood glucose (PBG) and area under curve (AUC) were determined. Whereas the maximum blood glucose concentration for each group was taken as PBG for the group, AUC was calculated using the relationship:

$$\begin{array}{ll} \text{AUC}\;\left(\text{mmol}/\text{L}\cdot \text{h}\right)\;= & \;\frac{{\text{BG}}_{0}{+\text{BG}}_{30\;\times \;0.5}}{2}\\ & +\frac{{\text{BG}}_{30}{+\text{BG}}_{60\times 0.5}}{2}\\ & +\frac{{\text{BG}}_{60}{+\text{BG}}_{120\times 1}}{2} \end{array}$$

Where BG represents the blood glucose concentration measured at time intervals 0, 30, 60 and 120 minutes.

#### Sucrose tolerance test

The sucrose tolerance test was carried out using the same procedure as for the determination of starch tolerance. However, in this test, sucrose at a dose of 4 g/kg body weight was used instead of starch.

#### Glucose tolerance test

The oral glucose tolerance test was also carried out using the same procedure as for the determination of starch tolerance, but glucose at a dose of 2 g/kg body weight was used instead of starch.

#### Confirmatory *in vivo* tests using streptozotocin-induced diabetic rats (SDRs)

This second set of tests also evaluated the effects of the active methanol extract (ME), fraction (NBF) and sub-fraction (SFI) on the tolerance of diabetic rats to orally administered starch, sucrose or glucose. In each test, 5 groups of rats (n = 6) were treated as follows. Groups 1-3 were treated with 1 g/kg each of ME, NBF and SFI, respectively and groups 4 (positive control) and 5 (negative control) were treated with acarbose (10 mg/kg) and an equivalent volume of distilled water (p.o.), respectively. As in earlier tests, 10 minutes after oral starch (3 g/kg)/sucrose (4 g/kg)/glucose (2 g/kg) treatment, blood glucose was measured at 0, 30, 60 and 120 min and used for PBG and AUC determinations similar as described above.

### Statistical analysis

Data are expressed as mean ± SEM. Analysis of variance (ANOVA) followed by post hoc analysis (Dunnett’s test) were used for data analysis using the SPSS statistical package, version 17.0. Differences at *P* < 0.05 were considered significant.

## Results

### *In vitro* inhibition of α-glucosidase activity

**Figure 1:**
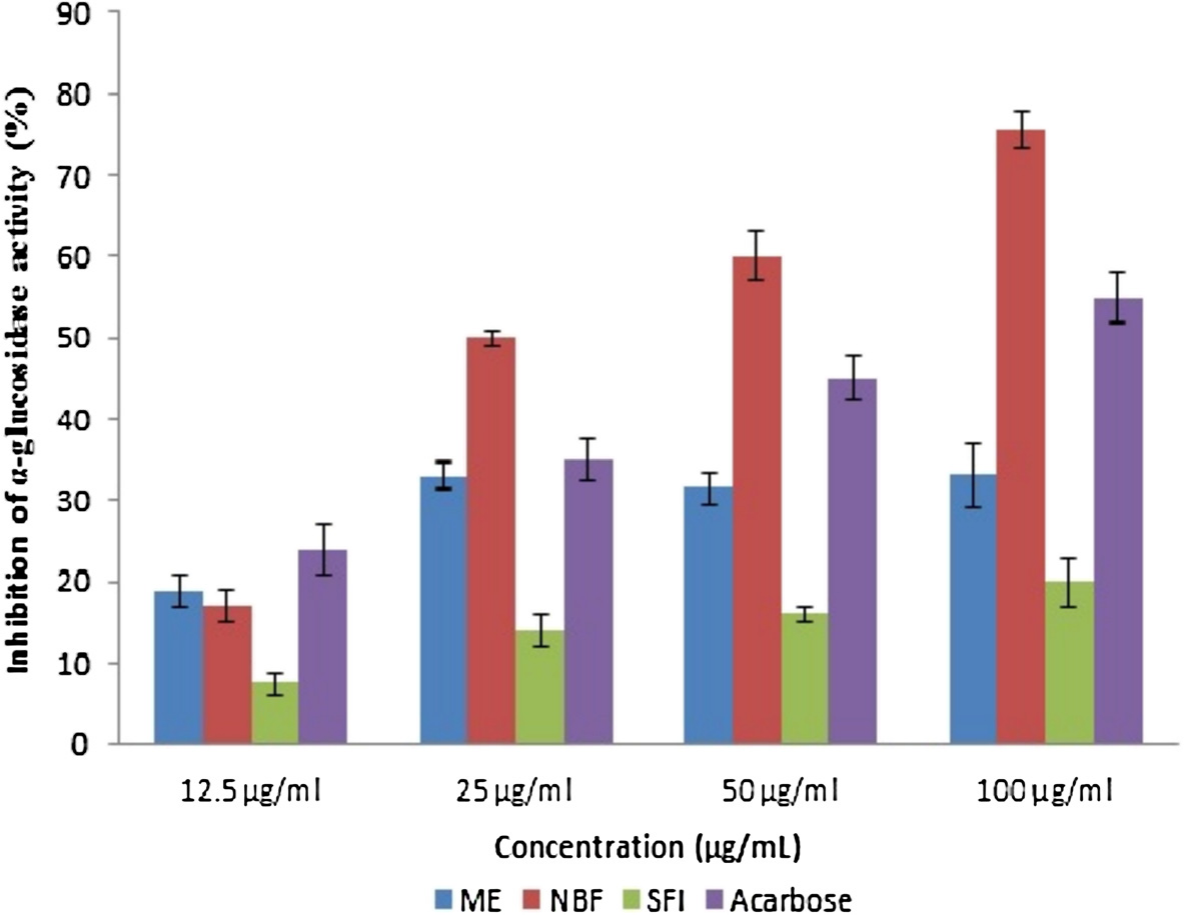
**Inhibitory effect of extract/fractions of *P*. *macrocarpa *fruit pericarp and acarbose on yeast α-glucosidase activity.** The test was performed in triplicate; values (% enzyme activity) are the mean ± SEM. ME: Methanol extract, NBF: n-Butanol fraction, SFI: Sub-fraction 1.

The methanol extract (ME), n-butanol fraction (NBF) and sub-fraction 1 (SFI) of *P*. *macrocarpa* potently inhibited α-glucosidase activity *in vitro* in a dose-dependent manner (Figure 1). At the highest concentration (100 μg/ml), NBF showed the highest percentage α-glucosidase inhibition of about 75%. This was 20% higher than that of acarbose (55%), a standard α-glucosidase inhibitor. The corresponding maximum inhibitory activities of the ME and SFI at the same concentrations were 32% and 20% respectively. This relative α-glucosidase inhibition was clearly indicated by the IC_50_ values (the concentration required for 50% inhibition) of each test analyte (Table 1). NBF with the highest inhibitory activity has lowest IC_50_ of 2.40 ± 0.23 μg/ml, closely followed by acarbose (3.40 ± 0.19 μg/ml). The methanol extract and SFI had IC_50_ values of 7.50 ± 0.15 μg/ml and 11.45 ± 0.28 μg/ml respectively.

**Table 1:** **IC**_**50 **_**values for yeast α-glucosidase and α-amylase inhibition**

**Analyte**	**Inhibitory concentration, IC**_**50 **_**(μg/ml)**	
	**α-glucosidase**	**α-amylase**
Methanol extract (ME)	7.50 ± 0.15	43.9 ± 0.19
n-butanol fraction (NBF)	2.40 ± 0.23	58.5 ± 0.13
Sub-fraction I (SFI)	11.45 ± 0.28	69.8 ± 0.25
Acarbose (ACAR)	3.40 ± 0.19	32.0 ± 0.30

### *In vitro* inhibition of α-amylase activity

**Figure 2:**
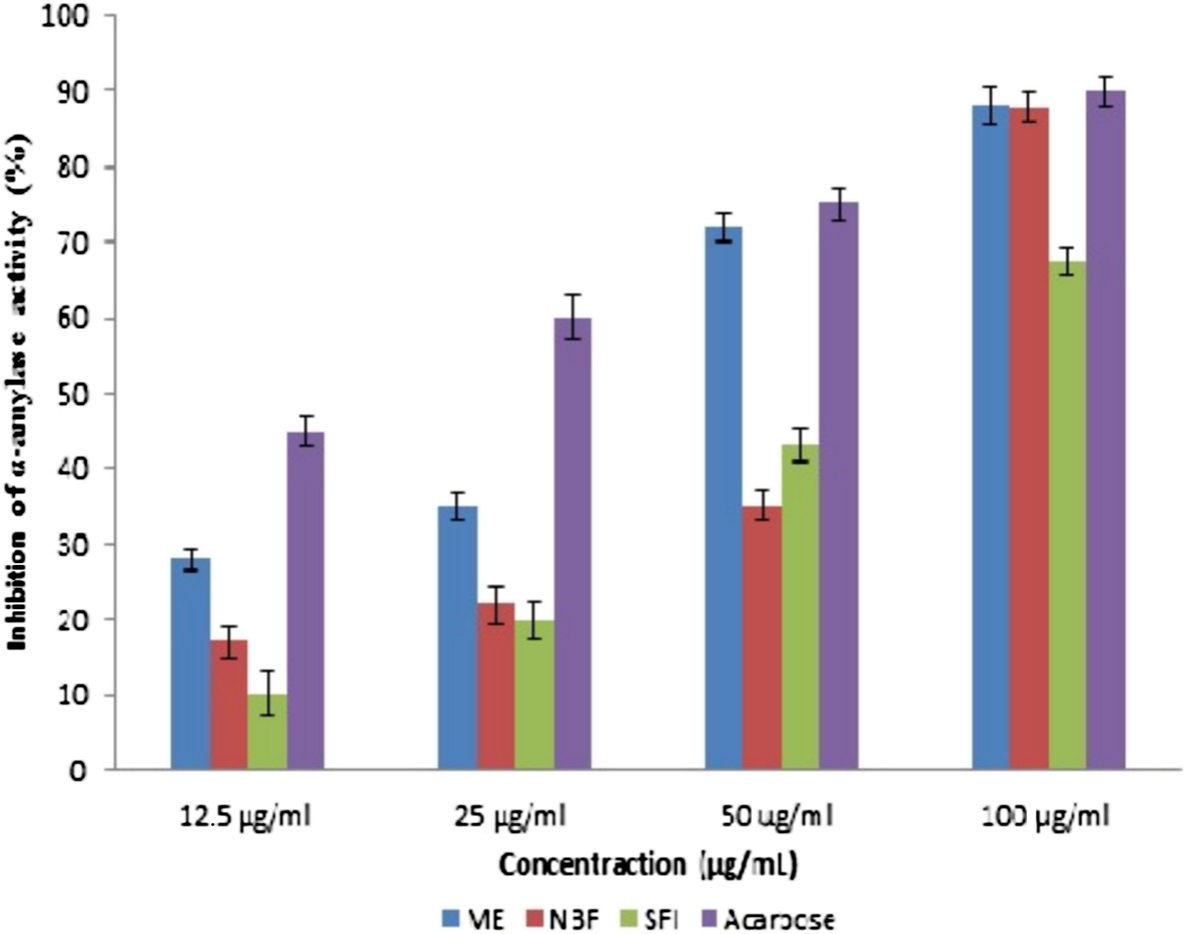
**Inhibitory effects of extract/fractions of *P*. *macrocarpa *fruit pericarp and acarbose on porcine α-amylase activity.** The test was performed in triplicate; values (% enzyme activity) are the mean ± SEM. ME: Methanol extract, NBF: n-Butanol fraction, SFI: Sub-fraction 1.

Figure 2 shows the % inhibition of α-amylase activity by ME, NBF and SFI along with the effect of acarbose. The standard drug, acarbose exerted the most potent inhibitory action against α-amylase of about 90% at 100 μg/ml. The inhibition by the extract and fractions were dependent on the concentrations/dose used. The methanol extract and NBF exerted similar peak inhibition activity of 87% while SFI inhibited α-amylase by 67% at the highest concentration used (100 μg/ml). The inhibition pattern also followed suit at lower concentrations.

### Effect of treatments on glucose tolerance tests in non-diabetic and diabetic rats

**Figure 3:**
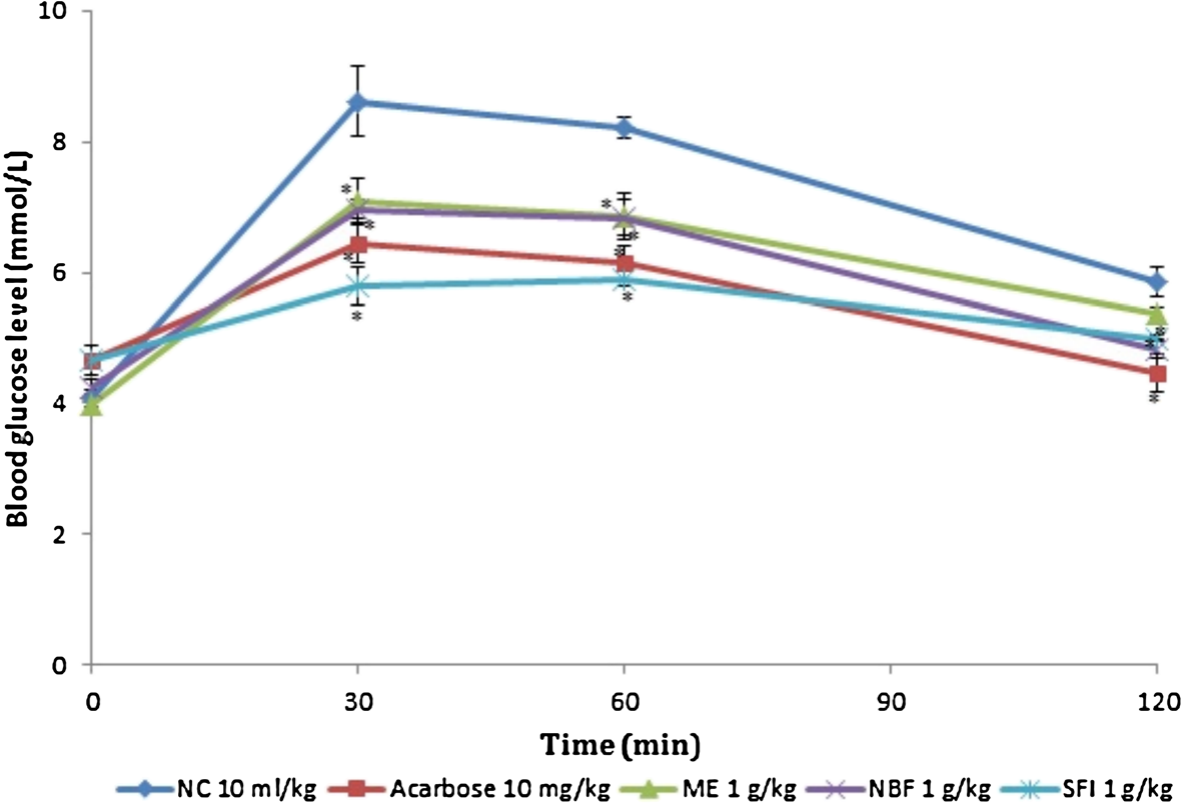
**Effects of treatments on glucose tolerance after oral glucose administration (2 g/kg) in non-diabetic rats (NDRs).** Values are the mean ± SEM (n = 6), *P < 0.05 vs. control. NC: Normal control, ACAR: Acarbose, ME: Methanol extract, NBF: n-Butanol fraction, SFI: Sub-fraction 1.

**Figure 4:**
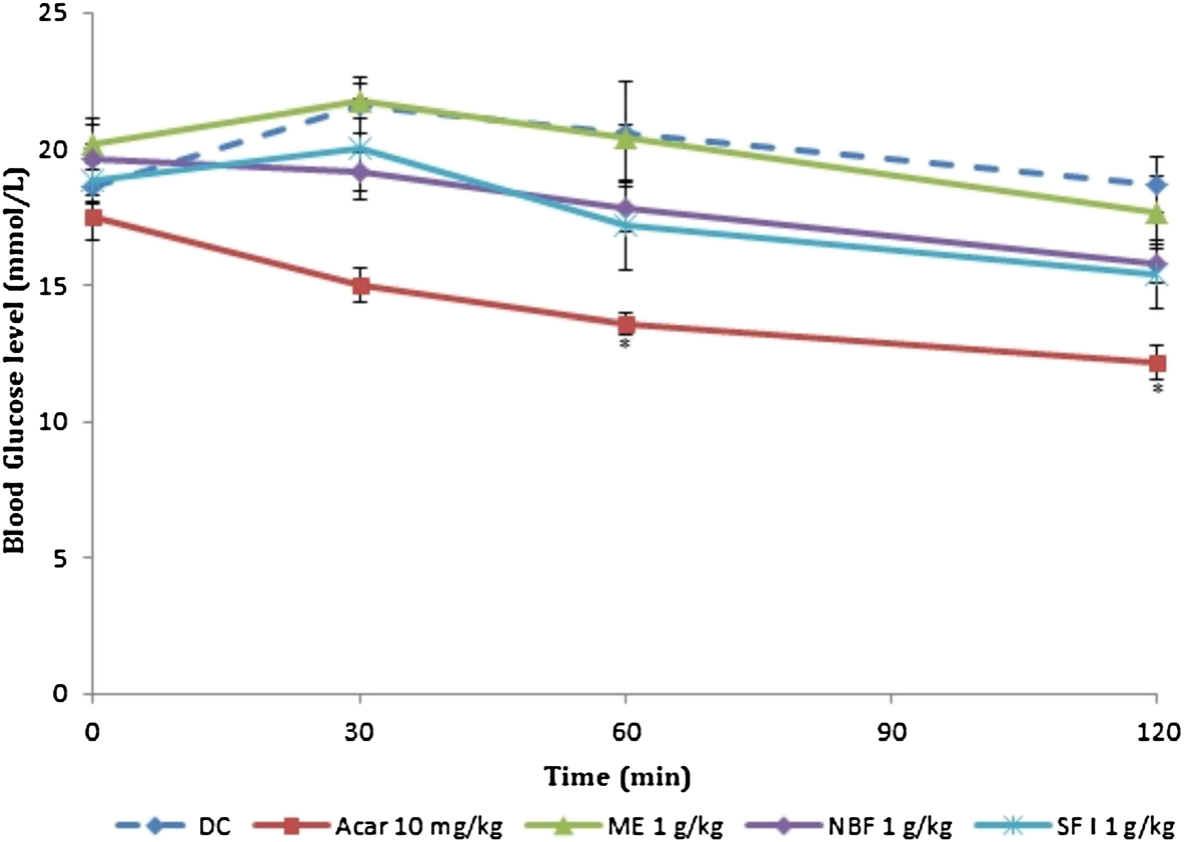
**Effect of treatments on glucose tolerance after oral glucose administration (2 g/kg) in streptozotocin-induced diabetic rats (SDRs).** Values are the mean ± SEM (n = 6) *P < 0.05 vs. control. DC: Diabetic control, ACAR: Acarbose, ME: Methanol extract, NBF: n-Butanol fraction, SFI: Sub-fraction 1.

Table 2 and Figures 3 and 4 show the effect of ME, NBF and SFI of *P*. *macrocarpa* on glucose tolerance in NDRs and SDRs. All treatments reduced the PBG in NDRs 30 minutes after oral glucose load, but only the reductions caused by SFI (30.34%) and acarbose (26.97%) were significant compared with the NCs. There was also a corresponding significant decrease of AUC in the 4 treatment groups compared with the control (*P* < 0.05), implying an onward flattening of the glucose tolerance curves (Figure 3). In the SDRs, the ME failed to exert any observable effect on the tolerance level following oral glucose loading. The NBF and SFI showed non-significant decreases in PBG levels (2.69% and 9.44%) and AUCs (7.21 and 13.40%) respectively. The control compound acarbose exerted a significant reduction of the PBG level (20.76%) and AUC (31.20%) in line with its known *in vivo* α-amylase inhibitory action.

**Table 2:** Effect of treatments on blood glucose (PBG) and area under the curve (AUC) after glucose loading (4 g/kg) in non-diabetic rats (NDRs) and STZ-diabetic rats (SDRs)

**Group**	**PBG (mmol/L)**	**% Reduction of PBG**	**AUC (mmol/L)**	**% Reduction of AUC**
Non-diabetic rats				
NC (vehicle)	8.9 ± 0.34		14.4 ± 0.32	
ACAR (10 mg/kg)	6.5 ± 0.32*	26.97	11.2 ± 0.44*	22.22
ME (1 g/kg)	7.4 ± 0.31	16.85	12.4 ± 0.48*	13.89
NBF (1 g/kg)	7.15 ± 0.18	19.66	12.08 ± 0.28*	16.11
SFI (1 g/kg)	6.20 ± 0.15*	30.34	11.04 ± 0.09*	23.33
Diabetic rats				
DC (vehicle)	21.92 ± 0.8		39.93 ± 1.75	
ACAR (10 mg/kg)	17.37 ± 0.66*	20.76	27.47 ± 0.9*	31.20
ME (1 g/kg)	22.05 ± 0.53	-	40.08 ± 0.74	-
NBF (1 g/kg)	21.33 ± 1.22	2.69	37.05 ± 1.6	7.21
SFI (1 g/kg)	19.85 ± 1.42	9.44	34.58 ± 2.34	13.40

Values are the mean ± SEM, n = 6, *P < 0.05 vs. control. *NC*: Normal control, *DC*: Diabetic control, *ACAR*: Acarbose, *ME*: Methanol extract, *NBF*: n-Butanol fraction, *SFI*: Sub-fraction 1.

### Effect of treatment on sucrose tolerance tests in non-diabetic and diabetic rats

**Figure 5:**
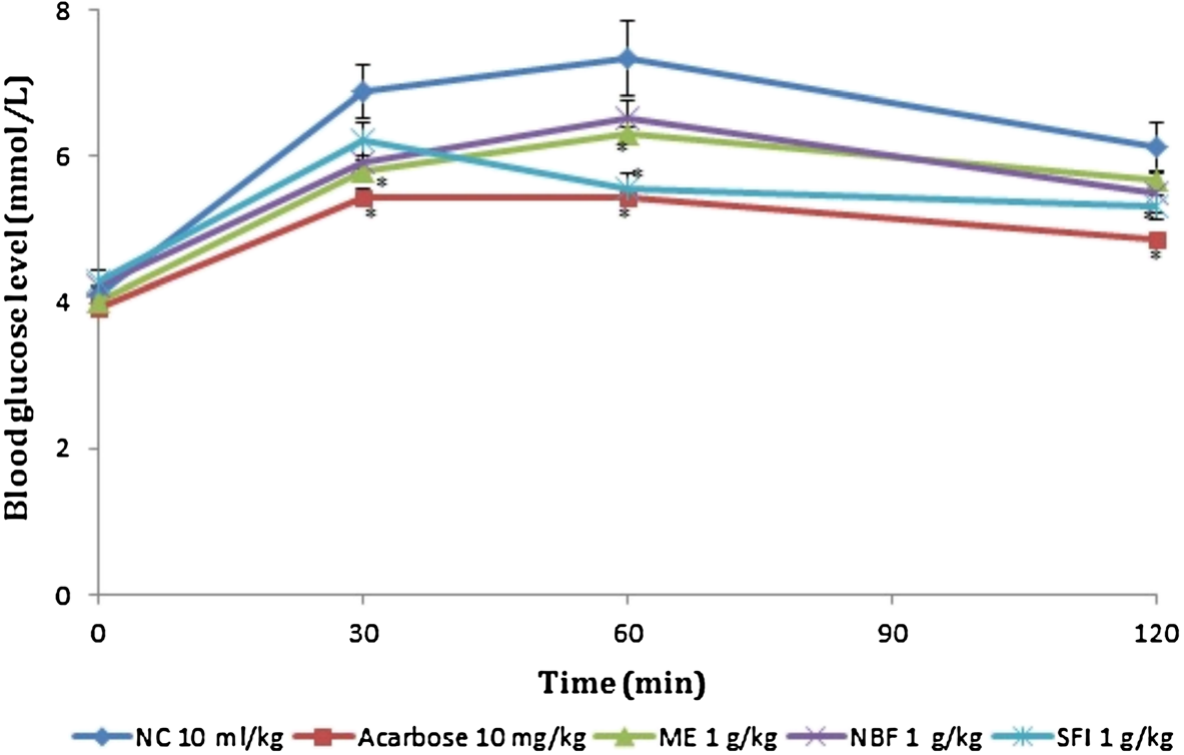
**Effect of treatments on sucrose tolerance after oral sucrose administration (4 g/kg) in non-diabetic rats (NDRs).** Values are the mean ± SEM (n = 6), *P < 0.05 vs. control. NC: Normal control, ACAR: Acarbose, ME: Methanol extract, NBF: n-Butanol fraction, SFI: Sub-fraction 1.

**Figure 6:**
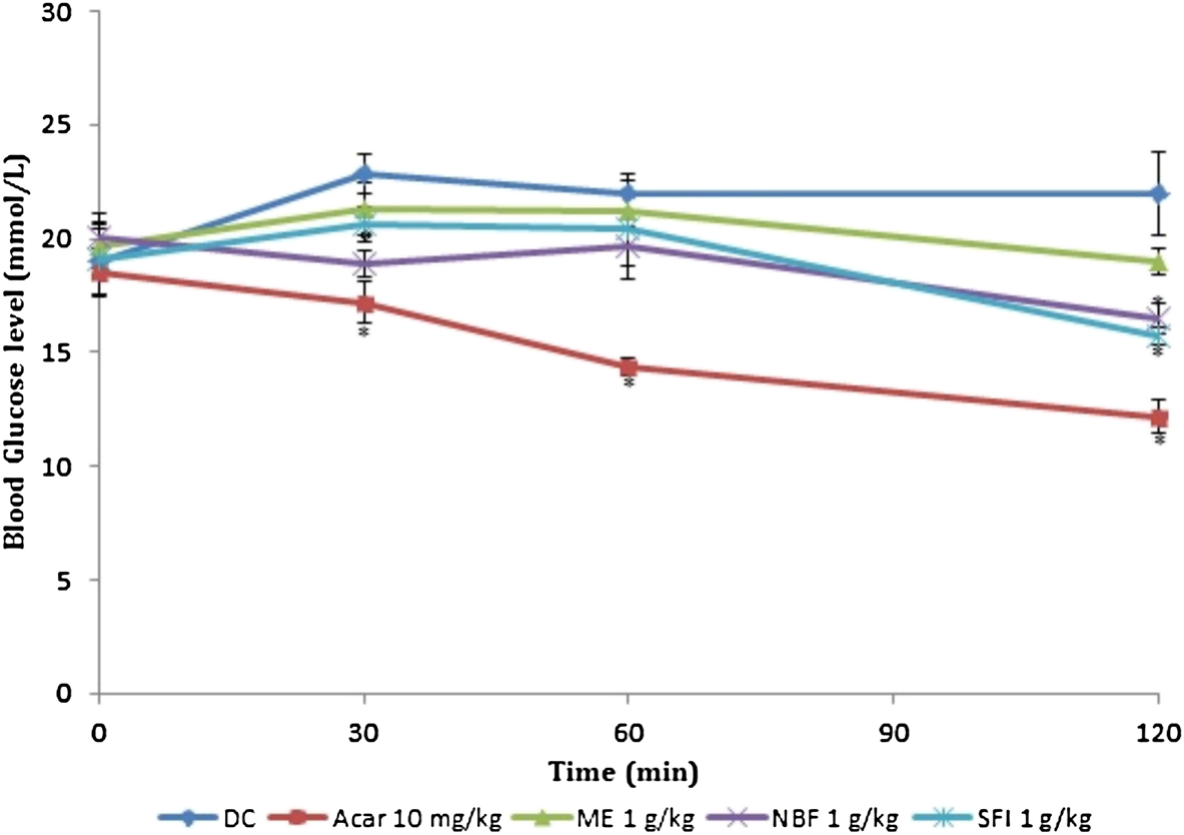
**Effect of treatments on sucrose tolerance after oral sucrose administration (4 g/kg) in streptozotocin-induced diabetic rats (SDRs).** Values are the mean ± SEM (n = 6), *P < 0.05 vs. control. DC: Diabetic control, ACAR: Acarbose, ME: Methanol extract, NBF: n-Butanol fraction, SFI: Sub-fraction 1.

The effects of *P*. *macrocarpa* extracts/fractions and acarbose on sucrose tolerance in NDRs and SDRs are shown in Table 3 and Figures 5 and 6. From the test results in NDRs, SFI alone demonstrated a strong inhibitory action against α-glucosidase *in vivo*, i.e. 17.28% and 15.57% reductions in PBG and AUC respectively, similar to the effect of acarbose (24.76% and 21.63%; *P* < 0.05). The ME and NBF showed weak activity against the enzyme *in vivo*. Results of the tolerance test in SDR revealed that NBF was the most effective at inhibiting α-glucosidase action *in vivo* i.e. 15.08% and 14.23% reductions in PBG and AUC respectively (*P* < 0.05). The *in vivo* inhibition corresponds to its high inhibitory activity observed in the *in vitro* α-glucosidase test. Although SFI was weak in reducing the PBG within the first 30 minutes, it effectively attenuated the tolerance curve over the 2 hours by 12.46% (*P* < 0.05). Acarbose was potent at reducing the PBG level (23.36%) and AUC (31.55%) significantly (*P* < 0.05).

**Table 3:** Effect of treatments on peak blood glucose (PBG) and area under the curve (AUC) after sucrose loading (4 g/kg) in non-diabetic rats (NDRs) and STZ-diabetic rats (SDRs)

**Group**	**PBG (mmol/L)**	**% Reduction of PBG**	**AUC (mmol/L)**	**% Reduction of AUC**
Non-diabetic rats				
NC (vehicle)	7.35 ± 0.41		13.04 ± 0.54	
ACAR (10 mg/kg)	5.53 ± 0.14*	24.76	10.22 ± 0.19*	21.63
ME (1 g/kg)	6.30 ± 0.10	14.29	11.46 ± 0.18	12.12
NBF (1 g/kg)	6.52 ± 0.18	11.29	11.65 ± 0.28	10.66
SFI (1 g/kg)	6.08 ± 0.14	17.28	11.01 ± 0.20*	15.57
Diabetic rats				
DC (vehicle)	24.14 ± 1.14		43.71 ± 1.10	
ACAR (10 mg/kg)	18.5 ± 0.96*	23.36	29.92 ± 0.78*	31.55
ME (1 g/kg)	21.5 ± 1.10	10.94	40.97 ± 1.66	6.27
NBF (1 g/kg)	20.53 ± 0.9	15.08	37.49 ± 1.45*	14.23
SFI (1 g/kg)	22.58 ± 1.45	6.46	38.27 ± 1.68*	12.46

Values are the mean ± SEM, n = 6, *P < 0.05 vs. control. *NC*: Normal control, *DC*: Diabetic control, *ACAR*: Acarbose, *ME*: Methanol extract, *NBF*: n-Butanol fraction, *SFI*: Sub-fraction 1.

### Effect of treatments on starch tolerance tests in non-diabetic and diabetic rats

**Figure 7:**
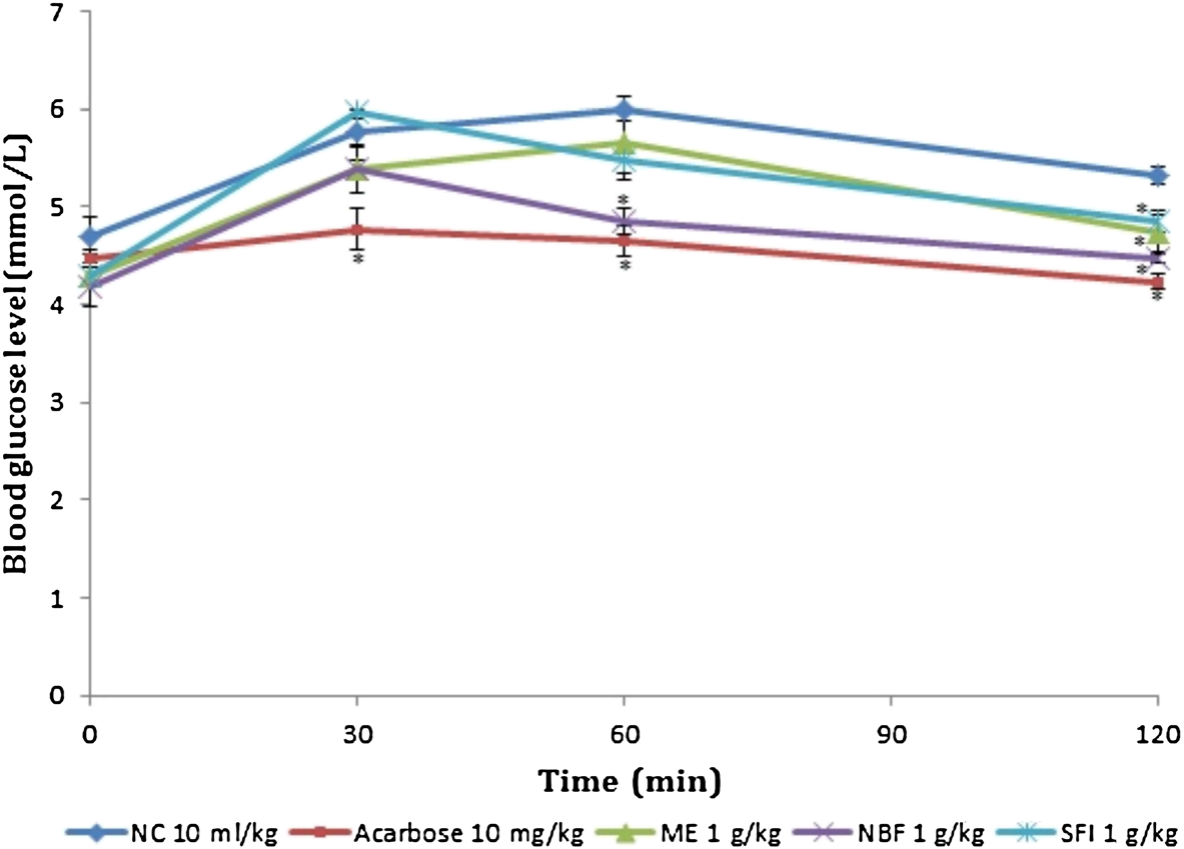
**Effect of treatments on starch tolerance after oral starch administration (3 g/kg) in non-diabetic rats (NDRs).** Values are the mean ± SEM (n = 6), *P < 0.05 vs. control. NC: Normal control, ACAR: Acarbose, ME: Methanol extract, NBF: n-Butanol fraction, SFI: Sub-fraction 1.

**Figure 8:**
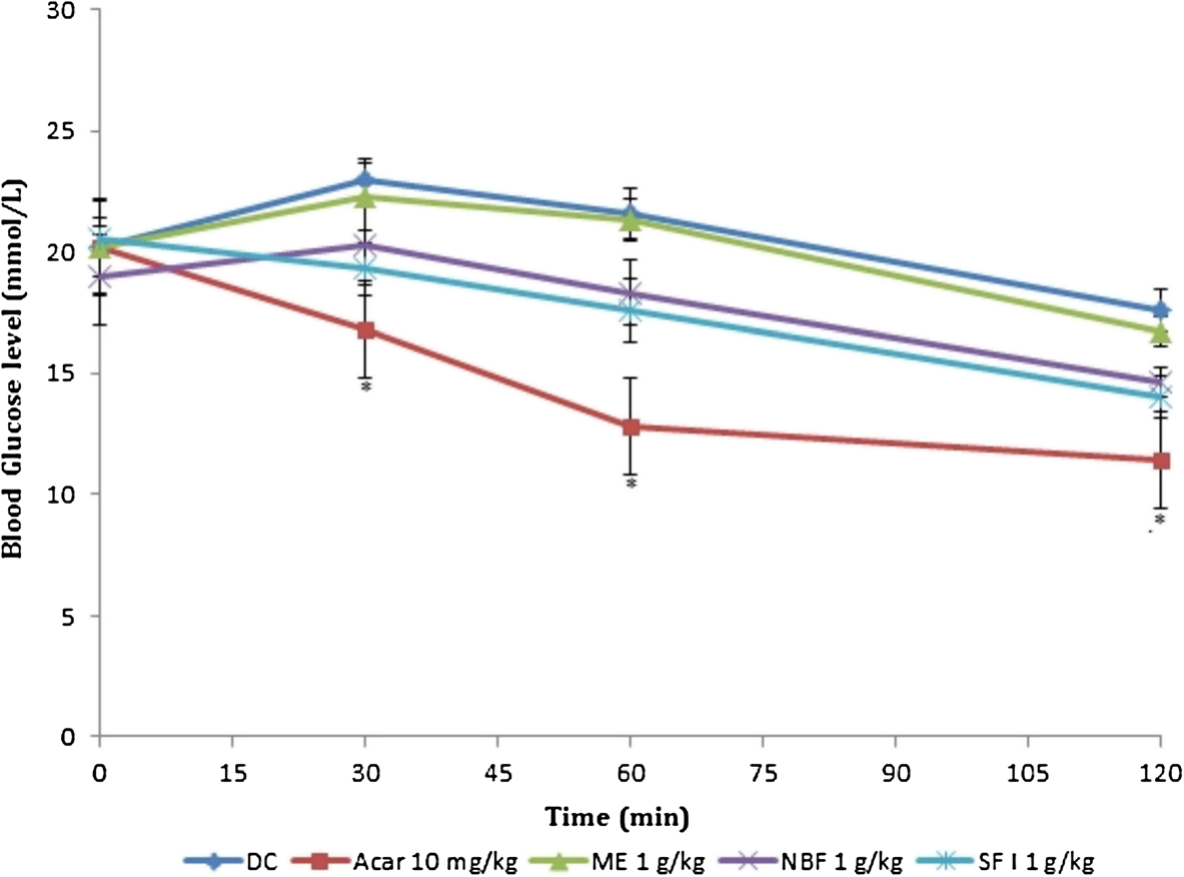
**Effect of treatments on starch tolerance after oral starch administration (3 g/kg) in streptozotocin-induced diabetic rats (SDRs).** Values are the mean ± SEM (n = 6), *P < 0.05 vs. control. DC: Diabetic control, ACAR: Acarbose, ME: Methanol extract, NBF: n-Butanol fraction, SFI: Sub-fraction 1.

In NDRs, both PBG and AUC were reduced by the 4 treatments (Table 4 and Figure 7). However, the reduction in PBG level was only significant in NBF and acarbose PBG treated groups and for the AUC in the acarbose treated group (*P* < 0.05). In SDRs, NBF and SFI decreased PBG and AUC non-significantly compared with the DC, whereas ME had no effect on the tolerance curve (Figure 8).

**Table 4:** Effect of treatments on peak blood glucose (PBG) and area under the curve (AUC) after starch loading (4 g/kg) in non-diabetic rats (NDRs) and STZ-diabetic rats (SDRs)

**Group**	**PBG (mmol/L)**	**% Reduction of PBG**	**AUC (mmol/L)**	**% Reduction of AUC**
Non-diabetic rats				
NC (vehicle)	6.12 ± 0.10		11.22 ± 0.17	
ACAR (10 mg/kg)	4.43 ± 0.11*	27.61	9.10 ± 0.19*	18.89
ME (1 g/kg)	5.78 ± 0.39	5.56	10.37 ± 0.50	7.58
NBF (1 g/kg)	5.18 ± 0.08*	15.36	9.61 ± 0.08	14.35
SFI (1 g/kg)	5.97 ± 0.05	2.45	10.58 ± 0.15	5.70
Diabetic rats				
DC (vehicle)	22.5 ± 1.02		41.2 ± 1.74	
ACAR (10 mg/kg)	18.7 ± 1.95	16.89	26.1 ± 2.8*	36.65
ME (1 g/kg)	22.6 ± 1.03	-	43.9 ± 2.99	-
NBF (1 g/kg)	21.1 ± 1.38	6.22	35.6 ± 2.32	13.59
SFI (1 g/kg)	19.1 ± 1.00	15.11	33.5 ± 1.5	18.69

Values are the mean ± SEM, n = 6, *P < 0.05 vs. control. *NC*: Normal control, *DC*: Diabetic control, *ACAR*: Acarbose, *ME*: Methanol extract, *NBF*: n-Butanol fraction, *SFI*: Sub-fraction 1.

## Discussion

α-Glucosidase inhibitors, a group of oral hypoglycaemic agents (OHA), have proven more useful and beneficial than other anti-diabetic drugs due to their exceptional benefits for management of post prandial hyperglycaemia (PPH). In terms of blood glucose lowering action, the α-glucosidase inhibitors are less effective compared with most other OHAs. Their singular advantage of averting the risk of PPH has made them most suitable in combination with other agents [17]. Therefore, the *in vitro* and *in vivo* α-glucosidase and α-amylase inhibitory effect of *P*. *macrocarpa*, a plant with evidence of strong empirical anti-diabetic properties, was investigated.

To our knowledge, the present study demonstrates for the first time the potent inhibitory activity of *P*. *macrocarpa* fruit extract and fractions against α-glucosidase and α-amylase *in vitro*. The n-butanol fraction had the highest inhibitory action against the two enzymes relative to the methanol extract and the sub-fraction. This effective inhibitory action of the n-butanol fraction can be correlated to its high mangiferin composition. An earlier LC-MS analysis of the three analytes revealed that mangiferin, a compound previously isolated from this plant, was the predominant bio-compound in *P*. *macrocarpa*, occurring at 9.52% in the methanol extract, 33.30% in the n-butanol fraction and 22.50% in the sub-fraction 1 [14]. Moreover, previously, mangiferin isolated from *Salacia species*, *Mangifera indica* and *Belamcanda chinensis* was also shown to exhibit strong inhibitory activities against α-glucosidase *in vitro*[18-20]. Additionally, the antidiabetic effect of mangiferin in streptozotocin-induced diabetic rats has been reported [21,22]. The involvement of mangiferin in the enzyme inhibition was also supported by the fact that the sub-fraction with the second highest content of mangiferin exerted the second highest inhibitory activity. Contrary to the expected result from activity-guided assays, the mangiferin content was found to be lower in SFI, the purer fraction, than NBF. The method required for processing, including the use of flash column and thin layer chromatography and treatment with several solvents, may have resulted in the degradation of mangiferin, accounting for its relatively low content and detection in the LC-MS assay.

The observed *in vitro* α-glucosidase and α-amylase inhibitory effects were replicated in the *in vivo* carbohydrate tolerance tests for the most part. The n-butanol fraction and sub-fraction 1 significantly reduced the AUC in streptozotocin-treated diabetic rats after oral sucrose administration. The extent of the reduction also corresponded to the mangiferin composition. A similar observation from a glucose tolerance test with extracts from young and old leaves and fruits of *P*. *macrocarpa* conducted in non-diabetic animals was reported by Sugiwati and co-workers [9,10]. Natural products with properties such as those identified in this study have the capacity to delay carbohydrate absorption into the blood stream by reversibly inhibiting the activity of carbohydrate digesting enzymes in the small intestinal brush border that are responsible for the breakdown of oligosaccharides and disaccharides into monosaccharides suitable for absorption [23]. Alternatively, some natural products may function by simply delaying the transfer of glucose from the stomach to the small intestine, the main site of glucose absorption (delayed gastric emptying rate) [24,25]. Consequently, these natural products avert the threat of hyperglycaemia after meals, and more importantly may provide glycaemic control without hyperinsulinaemia and body weight gain. Agents including conventional drugs that exploit any of the two targets to exert their anti-diabetic action are of particular interest for the management and prevention of type II diabetes mellitus.

## Conclusions

*P*. *macrocarpa* is a traditional medicinal plant with a long history of use as an anti-diabetic remedy in Asia Pacific countries. The present study, besides contributing to the scientific validation of the anti-diabetic properties of *P*. *macrocarpa*, suggests that attenuation of carbohydrate hydrolysis via inhibition of α-glucosidase and α-amylase activities is one mechanism of its hypoglycaemic and anti-hyperglycaemic therapeutic benefits. Moreover, mangiferin, a compound previously isolated from this plant may be responsible for the observed enzyme inhibition activity. Hence, *P*. *macrocarpa* is a prospective and veritable source of natural products for the management of type II diabetes mellitus.

## Competing interests

The authors declare that they have no competing interests.

## Authors’ contributions

RBA and NK carried out the animal experiments as part of their postgraduate research work. IJA and MA directed the experiments, articulated the results and drafted the manuscript. MZA and RM conceived the study, and participated in its design and coordination and helped to proof the draft manuscript. All authors read and approved the final manuscript.

## Pre-publication history

The pre-publication history for this paper can be accessed here:

http://www.biomedcentral.com/1472-6882/13/39/prepub
